# Foreword

**DOI:** 10.1093/pm/pnz078

**Published:** 2019-06-01

**Authors:** Dirk De Ridder

**Affiliations:** Neurosurgery, University of Otago, New Zealand

More than 2000 years ago, Epicurus (341–270 BC) stated that the pursuit of pleasure and absence of pain is the purpose of life. Thus, preventing or removing pain is one of the most beautiful and important aspects of life. Pain has been defined as an unpleasant sensory and emotional experience associated with actual or potential tissue damage, or described in terms of such damage [1]. Essentially, it means that when treating pain, both the sensory (=painfulness) and emotional (=suffering) components need to be addressed. Pain physicians are very competent in treating acute pain, usually related to actual tissue damage, yet when it comes to chronic pain, which is mostly neuropathic or fibromyalgic, the underlying pathophysiological mechanisms may be different and can be considered a centrally controlled imbalance between pain input and pain suppression [2] requiring an alternative approach.

Whereas acute pain can often be addressed pharmacologically and surgically, not every patient benefits from these interventions, and intractable patients may be offered neuromodulation. The first paper by Dr. Bates et al. nicely describes how a medical doctor can sequentially propose six different treatments for pain, ending with neuromodulation.

As a last resort, neuromodulation treatment should target both the sensory and emotional components of pain. Physiologically, painfulness and suffering are processed in parallel [3] via two different pathways/circuits ending in the somatosensory cortex for the sensory component and anterior cingulate cortex for the emotional component [3–5] of pain. The importance of this parallel processing cannot be overstated, as it means that when only treating the painfulness, the suffering or attentional component can still remain the same, even when painfulness is reduced dramatically.

The paper by Dr. Chakravarthy et al. describes how nonlinear burst stimulation, in contrast to tonic stimulation, modulates both the emotional and sensory pathways, explaining the pathophysiogical mechanism of action of burst stimulation, and this is clinically substantiated by the pooled analysis of real-world evidence in a second paper by the same authors. Dr. Kirketeig et al. critically review the literature supporting the use of burst stimulation and describe how burst -DR spinalcord stimulation is the only stimulation form that has been tested in a prospective placebo-controlled way, let alone multiple times, clearly demonstrating that it is a scientifically sound method of spinal cord modulation.

Yet neuromodulation is more than only spinal cord stimulation, and the dorsal root ganglion is a logical target for modulation of chronic pain input. In a paper by Dr. Esposito et al., the unique characteristics of the dorsal root ganglion are described, and a second paper by Dr. Antony et al. presents a review of the extraordinary results obtained by modulating this target.

This supplement thus gives a sound scientific summary of the pathophysiology-based mechanism of action and clinical outcomes for both burst-DR spinal cord stimulation and dorsal root ganglion stimulation, which is very helpful for any health care provider who wants to use evidence-based medicine to the benefit of their suffering patients.

Yet, finding new targets and new stimulation designs is only the first step in a revolution that has started within the field of neuromodulation. Recently, at the NANS conference in Las Vegas, Dr. Deer et al. presented new data looking at the benefits of drastically reducing electric dosage within patients [6]. This concept of providing patients with only the minimal dose necessary to achieve therapeutic effect appears promising and represents a paradigm shift in thinking. Along those lines, as a final paper in this compendium, Dr. Fishman addresses the future of neuromodulation. This future looks very bright, and might ultimately lead to an Epicurean world where “aponia” (=freedom of pain) is the rule.

Funding Source: None.

Conflict of Interest: Author has Intellectual Property on burst-DR as he invented this stimulation design.

Supplement sponsorship: This article appears as part of the supplement “Neuromodulation of the Spine and Nervous System” sponsored by Abbott.
